# Total tenderness score and pressure pain thresholds in persistent post-traumatic headache attributed to mild traumatic brain injury

**DOI:** 10.1186/s10194-022-01457-1

**Published:** 2022-08-08

**Authors:** Håkan Ashina, Haidar Muhsen Al-Khazali, Afrim Iljazi, Sait Ashina, Faisal Mohammad Amin, Henrik Winther Schytz

**Affiliations:** 1 Department of Anesthesia, Critical Care and Pain Medicine, Beth Israel Deaconess Medical Center, Harvard Medical School, Boston, MB USA; 2 Danish Headache Center, Department of Neurology, Rigshospitalet, Faculty of Health and Medical Sciences, University of Copenhagen, Copenhagen, Denmark; 3 Department of Neurorehabilitation / Traumatic Brain Injury, Rigshospitalet Glostrup, Copenhagen, Denmark; 4 BIDMC Comprehensive Headache Center, Departments of Neurology and Anesthesia, Critical Care, and Pain Medicine, Beth Israel Deaconess Medical Center, Harvard Medical School, Boston, MB USA; 5 Department of Clinical Medicine, Faculty of Health and Medical Sciences, University of Copenhagen, Copenhagen, Denmark

**Keywords:** Quantitative sensory testing, Concussion, Head trauma, Pathophysiology

## Abstract

**Objective:**

To investigate whether persistent post-traumatic headache attributed to mild traumatic brain injury (TBI) is associated with more pronounced pericranial tenderness and lower pressure pain thresholds (PPTs) in the head and neck region, compared with healthy controls.

**Methods:**

Patients with persistent post-traumatic headache (*n* = 100) and age- and gender-matched healthy controls (*n* = 100) were included between July 2018 and June 2019. Total tenderness score (TTS) was used to assess pericranial tenderness by bilateral manual palpation in eight muscles or tendon insertions. Summation was then used to calculate a TTS from 0 to 48 based on individual right- and left-sided scores; higher TTS score indicated more pronounced pericranial tenderness. PPTs were examined in m. temporalis and m. trapezius (upper and middle part) using an electronic pressure algometer that applies increasing blunt pressure at a constant rate.

**Results:**

The TTS score was higher in patients with persistent post-traumatic headache (median, 21; IQR, 12–31), compared with healthy controls (median, 10; IQR, 6–17; *P* < .001). PPTs were lower in patients with persistent post-traumatic headache than in controls in both the left-sided m. temporalis (mean ± SD, 157.5 ± 59.9 vs. 201.1 ± 65.2; *P* < .001) and right-sided m. temporalis (mean ± SD, 159.5 ± 63.8 vs. 212.3 ± 61.9; *P* < .001). Furthermore, patients with persistent post-traumatic headache also had lower left- and right-sided PPTs in the upper as well as middle part of m. trapezius, compared with healthy controls; all *P* values were .05 or less.

**Conclusions:**

Among patients with persistent post-traumatic headache, pericranial tenderness was more pronounced and PPTs in the head and neck region were lower than in healthy controls free of headache and mild TBI. Further research is needed to better understand the involvement of pericranial myofascial nociceptors in the disease mechanisms underlying post-traumatic headache.

**Supplementary Information:**

The online version contains supplementary material available at 10.1186/s10194-022-01457-1.

## Introduction

Persistent post-traumatic headache is a disabling neurologic disorder that is most often caused by mild traumatic brain injury (TBI) [1]. It is characterized by recurrent episodes of headache that vary in frequency, duration, and pain intensity [1]. The clinical features often resemble those of migraine and tension-type headache (TTH) [1–3], and it has therefore been posited that shared disease mechanisms might underlie these headache disorders [4, 5].

Experimental studies have shown that compared with healthy controls free of headache, people with migraine and TTH have increased pericranial tenderness and lower pressure pain thresholds (PPTs) in the head and neck region [6–11]. These results suggest that activation and sensitization of nociceptors in myofascial tissues might contribute to the genesis of cephalic pain [12–14]. In patients with persistent post-traumatic headache, three small studies reported lower cephalic PPTs compared with healthy controls [15–17]. Larger studies are however needed to ascertain what information can be gained from such experiments, because sensory testing might be influenced by variation in individuals’ perception of pain as well as the lack of uniformity in the methods applied. A well-established technique to examine pericranial tenderness has been coined ‘Total tenderness score’ (TTS) and involves bilateral manual palpation of muscles or tendon insertions in the head and neck region [14]. In regard to assessments of PPTs, quantitative sensory testing is best suited for the purpose, and an electronic pressure algometer can easily be used to apply increasing blunt pressure (at a constant rate) to the skin [9].

In this study, we examine whether pericranial tenderness is more pronounced in patients with persistent post-traumatic headache attributed to mild TBI, compared with age- and gender-matched healthy controls. We also investigate whether PPTs in the head and neck region are lower in patients with persistent post-traumatic headache than in healthy controls.

## Methods

The study protocol was approved by the independent ethics committee of the Capital Region of Denmark (H-18011477) and the Danish Data Protection Agency. All enrolled participants provided written informed consent. The study was conducted in accordance with the principles of the Declaration of Helsinki.

### Design and participants

The study design has been described in detail elsewhere [2, 18]. In brief, 100 patients with persistent post-traumatic headache and 100 age- and gender-matched healthy controls were enrolled. Patients were recruited from the outpatient clinic of the Danish Headache Center, patient support groups, neurological departments, and neurorehabilitation centers. Healthy controls were recruited via online public advertisement and posters placed at public institutions. The study is part of a larger parental study that is registered on ClinicalTrials.Gov (NCT03791515).

The study included patients aged 18 to 65 years who had a diagnosis of persistent post-traumatic headache attributed to mild TBI in accordance with the 3^rd^ edition of the International Classification of Headache Disorders (ICHD-3) and had sustained a mild TBI at least 12 months prior to enrollment [19]. Furthermore patients were allowed to have current use of preventive medications and report headache on the experimental day. Key exclusion criteria were any history of a primary headache disorder (except infrequent episodic TTH), medication-overuse headache, whiplash injury, and more than one sustained TBI. On the experimental day, patients were also excluded if they reported intake of acute medications within the past 24 h.

The control population included healthy individuals aged 18 to 65 years who had no history of a primary headache disorder (except infrequent episodic TTH), whiplash injury, TBI, neurological disorders, psychiatric disorders, and structural heart disease. Other eligibility criteria included no first-degree relatives with history of a primary headache disorder (except infrequent episodic TTH) and no daily intake of medications other than oral contraceptives.

### Procedures

For patients with persistent post-traumatic headache, a semi-structured interview was performed by trained personnel to collect data on demographics, medical history, and full clinical course. Electronic medical records were screened to assess whether participants had a history of physical or mental illness. Conditions associated with persistent post-traumatic headache were evaluated using the following self-report instruments: the 19-item Pittsburg Sleep Quality Index (PSQ-I) to evaluate quality of sleep and the 14-item Hospital Anxiety and Depression Scale (HADS) to screen for anxiety and/or depression [20, 21]. The range of the scales and interpretation of the results in the present study populations have been described elsewhere [18]. In addition, the presence and severity of cutaneous allodynia was assessed using the 12-item Allodynia Symptom Checklist (ASC-12) [22]. The range of the scale is from 0 to 24, with scores of 3 to 5 defined as mild cutaneous allodynia while scores of 5 to 8 are classified as moderate cutaneous allodynia. Scores of ≥ 9 are defined as severe cutaneous allodynia.

#### Total tenderness score (TTS)

TTS is used to assess pericranial tenderness in the following eight muscles or tendon insertions: m. frontalis, m. masseter, m. pterygoideus lateralis, m. sternocleidomastoideus, m. temporalis, m. trapezius, mastoid process, and nuchal lines of the occipital bone. A 4-point scale (0 to 3) is used to score the presence and severity of tenderness in each muscle or tendon insertion. The individual right-sided and left-sided scores are then used to calculate by summation a TTS from 0 to 48.

The methodology used to acquire TTS data has been described in detail elsewhere [14, 23, 24]. In brief, one investigator (A.I.) was trained in bilateral manual palpation of each assessed muscle or tendon insertion with small rotating movements using the pulp of the index finger and middle finger. A palpometer was then used to ensure that the investigator was able to exert a standardized pressure of 80 kPa to all assessed muscles or tendon insertions.

#### Pressure pain threshold (PPT)

PPTs were measured in m. temporalis and m. trapezius (upper and middle part). The measurements were performed by one investigator (A.I.) in an undisturbed examination room. An electronic pressure algometer was used and held perpendicular to the skin in order to apply increasing blunt pressure at a constant rate. The circular stimulation probe was 1 cm^2^, and the pressure loading rate was 50 kPa/s. The latter was controlled in real-time using an electronic visual display. Three measurements were performed at each location to calculate a mean PPT value, with an interval of at least one minute between each measurement. Counterpressure was applied to support and keep the participants’ head still. The PPT was defined as the pressure at which the participant pressed a button to indicate that the sensation has changed from pressure to pain.

### Statistical analysis

Demographics and clinical characteristics were summarized with descriptive statistics. The Shapiro–Wilk test was used to assess whether TTS and PPT data followed a normal distribution. The median value with interquartile ranges (IQR) was presented for TTS data while the mean ± standard deviations (SD) was used to describe PPT data. To compare differences between two unpaired groups, the unpaired t test or Mann Whitney U test was used as appropriate, and two-tailed *P*-values were calculated. Correlation coefficients, *r*, were calculated using the Spearman’s rank correlation coefficient test. The level of significance was alpha = 0.05. All analyses were performed with R (v4.1.0).

## Results

### Participants

A total of 100 patients with persistent post-traumatic headache and 100 age- and gender-matched healthy controls were enrolled into this study. Characteristics of the study population and control group are presented in Table 1. Patients’ mean age was 36.0 ± 11.7 years, and 83% were women. The mean number of monthly headache days was 25.4 ± 7.1, and 91% had a migraine-like headache phenotype. Conditions associated with persistent post-traumatic headache were poor quality of sleep (85%), recurrent episodes of neck pain (78%), probable to high risk of anxiety (52%), and probable to high risk of depression (42%). In addition, the proportion of patients with mild, moderate, or severe cutaneous allodynia was 23%, 17%, and 6%, respectively. Additional characteristics of the patient population have been published elsewhere and are also summarized in Table 2 [2, 18].

**Table 1:** Summary of the study populations

Variable	Persistent PTH (*n* = 100)	Healthy controls (*n* = 100)
**Age**, mean (SD), y	36.0 (11.7)	35.8 (11.3)
**Male/female**, %	17/83	18/82
**Height**, mean (SD), cm	171.3 (8.2)	170.9 (8.7)
**Weight**, mean (SD), kg	72.1 (14.4)	71.2 (14.2)
**BMI** mean (SD), kg/m^2^	24.5 (4.1)	24.3 (3.7)

*PTH* Post-Traumatic Headache, *BMI* Body Mass Index

**Table 2:** Characteristics of the patient population

Characteristics	Persistent PTH (*n* = 100)
**Employment status**, %	
Full-time employed	37
Part-time employed	42
Unemployed	21
**Education**	
Years of education, mean (SD), y	15.6 (2.8)
No education besides completion of secondary school or high school, %	17
Skilled labor, %	25
Bachelor´s degree, %	38
Higher education, %	20
**Disease duration**, mean (SD), months	49.0 (37.9)
**Headache phenotypes**, %	
Chronic migraine-like	61
Episodic migraine-like	1
Episodic migraine-like combined with chronic TTH-like	25
Episodic migraine-like combined with frequent TTH-like	2
Episodic migraine-like combined with infrequent TTH-like	2
Chronic TTH-like	9
**Headache frequency**, mean (SD)	
Yearly headache days	307.9 (86.9)
Monthly headache days	25.4 (7.1)
**Migraine-like headache phenotype** (*n* = 91)	
Monthly headache days, mean (SD)	24.9 (127.1)
Monthly migraine days, mean (SD)	14.5 (10.4)
Aura, %	8
Headache quality, %	
Throbbing	20
Pressing	25
Stabbing	3
Combined (throbbing and pressing)	49
Other headache quality	2
**Current use of preventive medications**, %	55
**ASC-12 scores**, mean score (SD)	3.1 (3.5)
None, %	54
Mild allodynia, %	23
Moderate allodynia, %	17
Severe allodynia, %	6
**Global PSQ-I scores**, mean score (SD)	8.9 (3.9)
Poor quality of sleep, %	85
**HADS anxiety scores**, mean score (SD)	8.3 (4.6)
Probable or high risk of anxiety, %	52
Probable risk of anxiety, %	19
High risk of anxiety, %	33
**HADS depression scores**, mean score (SD)	6.6 (3.9)
Probable or high risk of depression, %	42
Probable risk of depression, %	30
High risk of depression, %	12

*PTH* Post-Traumatic Headache, *ASC-12* 12-item Allodynia Symptom Checklist, *PSQ-I* Pittsburg Sleep Quality Index, *HADS* Hospital Anxiety and Depression Scale

### Total tenderness score

The median number of TTS was 21 (IQR, 12–31) in patients with persistent post-traumatic headache and 10 (IQR, 6–17) in healthy controls, and the difference was significant (*P* < 0.001). Compared with controls, patients with post-traumatic headache also had higher left-sided TTS (*P* < 0.001) and higher right-sided TTS (*P* < 0.001).

Among patients, TTS was correlated with age (*r*_*s*_ = -0.31, *P* = 0.002), ASC-12 scores (*r*_*s*_ = 0.28, *P* = 0.005), and HADS anxiety scores (*r*_*s*_ = 0.20, *P* = 0.05), (Table 3). No correlations were observed between TTS and monthly headache days (*r*_*s*_ = -0.12, *P* = 0.20), Global PSQ-I scores (*r*_*s*_ = 0.13, *P* = 0.21), HADS depression scores (*r*_*s*_ = -0.01, *P* = 0.89), and headache intensity on the assessment day (*r*_*s*_ = -0.07, *P* = 0.52), (Table 3).

**Table 3:** Correlation of total tenderness score with demographics and clinical characteristics in patients with persistent post-traumatic headache and healthy controls

**Variable**	**Persistent PTH (*n* = 100)**		**Healthy controls (*n* = 100)**	
	Correlation coefficient	*P* value	Correlation coefficient	*P* value
Age	-0.31	.002	0.01	.91
Monthly headache days	-0.12	.20	NA	NA
ASC-12 scores	0.28	.005	NA	NA
Global PSQ-I scores	0.13	.21	0.13	.20
HADS anxiety scores	0.20	.05	0.03	.74
HADS depression scores	-0.01	.89	-0.04	.67

*PTH* Post-Traumatic Headache, *ASC-12* 12-item Allodynia Symptom Checklist, *PSQ-I* Pittsburg Sleep Quality Index, *HADS* Hospital Anxiety and Depression Scale

### Pressure pain thresholds

For the left-sided m. temporalis, mean PPT values were lower in patients with persistent post-traumatic headache (157.5 ± 59.9), compared with healthy controls (201.1 ± 65.2; *P* < 0.001). Mean PPT values obtained from the right-sided m. temporalis were also lower in patients (159.5 ± 63.8), compared with healthy controls (212.3 ± 61.9; *P* < 0.001). There were no correlations for PPT values from both the left- and right-sided m. temporalis of patients with any of the following variables: age, monthly headache days, ASC-12 scores, Global PSQ-I scores, HADS anxiety scores, HADS depression scores, and headache intensity on the assessment day (Table 4).

**Table 4:** Correlations of mean pressure pain threshold scores in m. temporalis in patients with persistent post-traumatic headache

**Variable**	**Left side**		**Right side**	
	Correlation coefficient	*P* value	Correlation coefficient	*P* value
Age	0.04	.69	0.03	.78
Monthly Headache Days	-0.08	.42	-0.10	.33
ASC-12 Scores	-0.12	.22	-0.16	.10
Global PSQ-I Scores	-0.02	.88	-0.05	.59
HADS Anxiety Scores	-0.10	.31	-0.09	.39
HADS Depression Scores	-0.06	.58	-0.06	.58

*ASC-12* 12-item Allodynia Symptom Checklist, *PSQ-I* Pittsburg Sleep Quality Index, *HADS* Hospital Anxiety and Depression Scale

For the upper part of the left-sided m. trapezius, mean PPT values were lower in patients (241.6 ± 104.2) than in healthy controls (308.0 ± 114.7; *P* < 0.001). Mean PPT values obtained from the corresponding right-sided m. trapezius were also lower in patients with persistent post-traumatic headache (277.1 ± 117.0), compared with the control group (317.5 ± 123.0; *P* = 0.01). Among patients, correlations were observed between age and PPT values in the upper part of both the left-sided (*r*_*s*_ = 0.21, *P* = 0.04) and right-sided m. trapezius (*r*_*s*_ = 0.19, *P* = 0.05), (Table 5). There were no correlations for left- or right-sided PPT values with monthly headache days, ASC-12 scores, Global PSQ-I scores, HADS anxiety scores, HADS depression scores, and headache intensity on the assessment day (Table 5).

**Table 5:** Correlations of mean pressure pain threshold scores in upper part of m. trapezius in patients with persistent post-traumatic headache

**Variable**	**Upper part of m. trapezius**				**Middle part of m. trapezius**			
	Left side		Right side		Left side		Right side	
	Correlation coefficient	*P* value	Correlation coefficient	*P* value	Correlation coefficient	*P* value	Correlation coefficient	*P* value
Age	0.21	.04	0.19	.05	0.16	.11	0.18	.07
Monthly headache days	0.01	.95	0.02	.83	-0.03	.77	0.03	.79
ASC-12 scores	-0.14	.17	-0.14	.17	-0.12	.22	-0.19	.06
Global PSQ-I scores	0.10	.33	-0.04	.66	0.05	.65	-0.10	.37
HADS anxiety scores	0.09	.35	-0.01	.89	0.09	.38	-0.12	.24
HADS depression scores	0.11	.29	-0.02	.84	0.16	.11	-0.02	.81

*ASC-12* 12-item Allodynia Symptom Checklist, *PSQ-I* Pittsburg Sleep Quality Index, *HADS* Hospital Anxiety and Depression Scale

Mean PPT values obtained from the middle part of the left-sided m. trapezius were lower in patients with persistent post-traumatic headache (237.2 ± 107.5), compared with healthy controls (281.0 ± 109.2; *P* = 0.003). Patients also had lower mean PPT values in the corresponding right-sided m. trapezius (251.0 ± 111.0), compared with the control population (311.6 ± 131.3, *P* < 0.001). No correlations were observed for PPT values from the middle part of both the left- and right-sided m. trapezius of patients with any of the following variables: age, monthly headache days, ASC-12 scores, Global PSQ-I scores, HADS anxiety scores, HADS depression scores, and headache intensity on the assessment day (Table 5).

## Discussion

The present study shows that pericranial tenderness is more pronounced in patients with persistent post-traumatic headache than in age- and gender-matched healthy controls free of headache and mild TBI. In addition, we found that thresholds for pressure pain stimuli are lower in the head and neck region of patients, compared with healthy controls. These findings support our primary hypotheses, and it is thus evident that nociceptive thresholds to mechanical stimuli are lowered in patients who develop persistent post-traumatic headache following mild TBI. Furthermore, the presence of pericranial tenderness might indicate a myofascial contribution to the genesis of cephalic pain in patients with persistent post-traumatic headache. In this context, it is possible that myofascial pain in post-traumatic headache is the result of peripheral sensitization of nociceptors in myofascial tissues that are located in the head and neck region. Another explanation might involve central sensitization of second-order neurons at the level of the trigeminal cervical complex. In the latter instance, it is generally assumed that central sensitization is induced and, in part, maintained by prolonged nociceptive input from pain-sensitive structures outside of the CNS (e.g. meninges, myofascial tissues).

To our knowledge, this is the first study to demonstrate increased pericranial tenderness in patients with persistent post-traumatic headache, compared with healthy controls. Our observation accords well with the results of a recent meta-analysis [10], in which the authors concluded that pericranial tenderness is more pronounced in patients with migraine and TTH than in healthy controls. Moreover, the present correlation of higher TTS with younger age is congruent with previous findings in a population-based sample of people with chronic TTH [8]. However, this observation does not seem specific to people with headache disorders, as pericranial tenderness was more pronounced in younger adults from the general population [25]. On another note, we also found positive correlations of TTS with cutaneous allodynia scores and anxiety scores. However, these correlations should be interpreted with caution, as they are considered weak (*r*_*s*_ < 0.5) and have not previously been investigated for other headache disorders, such as migraine and TTH.

A few small studies have previously assessed PPTs in patients with post-traumatic headache [15–17]. In one study [15], the authors included 17 patients with persistent post-traumatic headache attributed to TBI (mild, moderate, or severe), 11 patients with TBI and free of headache, and an unspecified number of healthy controls. PPTs were assessed in regions of the head and the hand. The authors found that PPTs in regions of the head were lower in patients with persistent post-traumatic headache, compared with both TBI patients free of headache and healthy controls. No differences were however found for PPTs in the hand. Based on these findings, we can infer that PPTs are likely only lowered in the head region (and probably neck region) in patients with persistent post-traumatic headache. This aligns well with the conclusions made in a recent meta-analysis of PPTs in patients with migraine, compared with healthy controls [26]. The authors concluded that PPTs were lower in patients with migraine than in healthy controls when pressure stimuli were applied to the head and neck region but not when applied below the neck region (i.e. rest of the body).

### Limitations

This study has several limitations. First, the lack of blinding might result in observer bias. We did however ensure that all measurements were performed by the same investigator (A.I.). Second, we did not assess PPTs in body regions below the head and neck and, consequently, we cannot exclude that PPTs are indeed only lower in the head and neck region in patients with persistent post-traumatic headache. Although it should be noted that no differences in PPTs below the head and neck region have been observed when patients with persistent post-traumatic headache and migraine have been compared with healthy controls [15, 26]. Third, patients were mostly enrolled from the outpatient clinic of the Danish Headache Center. These patients are more adversely affected, compared with a representative sample of people with persistent post-traumatic headache in the general population. Fourth, we did not perform blood sampling and subsequent measurements of sex hormones that might be associated with the responsiveness to pain stimuli [27]. Lastly, it is critical to note that we did not include a control group of participants with mild TBI who were free of headache. Additional research is therefore warranted to examine whether pericranial tenderness and lowered PPTs in the head and neck region are attributed to persistent post-traumatic headache, mild TBI, or both. Such insights will, in turn, also facilitate an improved understanding of shared and distinct disease mechanisms underlying migraine and persistent post-traumatic headache [28–30].

## Conclusions

Among patients with persistent post-traumatic headache attributed to mild TBI, pericranial tenderness was more pronounced and PPTs in the head and neck region were lower than in healthy controls free of headache and mild TBI. Myofascial tissues might therefore represent an important, but underappreciated, source of cephalic pain. Further research is needed to ascertain the involvement of pericranial myofascial tissue in the neurobiologic underpinnings of post-traumatic headache.

## Availability of data and materials

Qualified researchers can request access to patient-level data and related study documents, including the study protocol. Patient-level data will be deidentified and study documents will be redacted to protect the privacy of study participants.

## Supplementary Information

**Additional file 1:** **Supplemental Figure 1.** Total tenderness score in 100 patients with persistent post-traumatic headache and 100 healthy controls. **Supplemental Figure 2.** Pressure pain thresholds in m. temporalis (left- and right-sided) of 100 patients with persistent post-traumatic headache and 100 healthy controls. **Supplemental Figure 3.** Pressure pain thresholds in the upper part of m. trapezius (left- and right-sided) in 100 patients with persistent post-traumatic headache and 100 healthy controls. **Supplemental Figure 4.** Pressure pain thresholds in the middle part of m. trapezius (left- and right-sided) in 100 patients with persistent post-traumatic headache and 100 healthy controls.

## Abbreviations

TBI: Traumatic brain injury
TTH: Tension-type headache
PPT: Pressure pain threshold
ICHD-3: International Classification of Headache Disorders, 3rd edition
PSQ-I: Pittsburg Sleep Quality Index
HADS: Hospital Anxiety and Depression Scale
ASC-12: 12-Item Allodynia Symptom Checklist
IQR: Interquartile range
SD: Standard deviation
