# Supplementary material for: Total tenderness score and pressure pain thresholds in persistent post-traumatic headache attributed to mild traumatic brain injury
Source: J Headache Pain. 2022 Aug 8;23(1):96. doi: 10.1186/s10194-022-01457-1 (PMC9358841; doi:10.1186/s10194-022-01457-1)
Supplement: Supplementary file 1 — Additional file 1: Supplemental Figure 1. Total tenderness score in 100 patients with persistent post-traumatic headache and 100 healthy controls. Supplemental Figure 2. Pressure pain thresholds in m. temporalis (left- and right-sided) of 100 patients with persistent post-traumatic headache and 100 healthy controls. Supplemental Figure 3. Pressure pain thresholds in the upper part of m. trapezius (left- and right-sided) in 100 patients with persistent post-traumatic headache and 100 healthy controls. Supplemental Figure 4. Pressure pain thresholds in the middle part of m. trapezius (left- and right-sided) in 100 patients with persistent post-traumatic headache and 100 healthy controls. [file 10194_2022_1457_MOESM1_ESM.docx]

**Supplemental Figure 1. Total tenderness score in 100 patients with persistent post-traumatic headache and 100 healthy controls.**


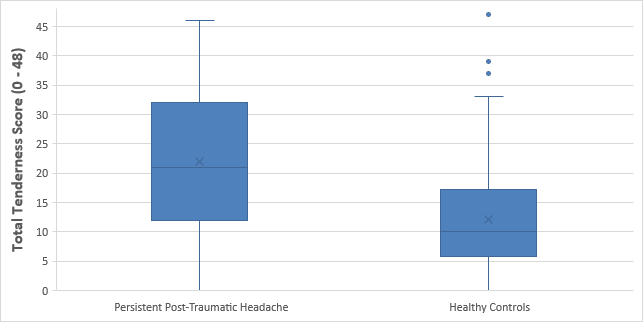


**Supplemental Figure 2. Pressure pain thresholds in m. temporalis (left- and right-sided) of 100 patients with persistent post-traumatic headache and 100 healthy controls.**

PTH: Post-traumatic headache; HC: Healthy controls.

**Supplemental Figure 3. Pressure pain thresholds in the upper part of m. trapezius (left- and right-sided) in 100 patients with persistent post-traumatic headache and 100 healthy controls.**

PTH: Post-traumatic headache; HC: Healthy controls.

**Supplemental Figure 4. Pressure pain thresholds in the middle part of m. trapezius (left- and right-sided) in 100 patients with persistent post-traumatic headache and 100 healthy controls.**

PTH: Post-traumatic headache; HC: Healthy controls.
